# CPT1 deficiency blocks autophagic flux to promote lipid accumulation induced by co-exposure to polystyrene microplastic and cadmium

**DOI:** 10.3389/fphar.2024.1533188

**Published:** 2025-01-06

**Authors:** Zhixuan Chen, Huayi Qu, Jian Sun, Tao Wang, Yan Yuan, Jianhong Gu, Jianchun Bian, Zongping Liu, Hui Zou

**Affiliations:** ^1^ College of Veterinary Medicine, Yangzhou University, Yangzhou, China; ^2^ Joint International Research Laboratory of Agriculture and Agri-Product Safety, The Ministry of Education of China, Yangzhou University, Yangzhou, Jiangsu, China; ^3^ Jiangsu Co-innovation Center for Prevention and Control of Important Animal Infectious Diseases and Zoonoses, Yangzhou, China

**Keywords:** autophagy, cadmium, liver, lipid metabolism, polystyrene microplastics

## Abstract

**Introduction:**

Cadmium (Cd) and polystyrene microplastics (PS-MPs), two ubiquitous environmental contaminants, produce unique synergistic toxicity when co-existing. Key unanswered questions include specific effects on liver function and potential mechanisms.

**Methods:**

In this study, C57BL/6 mice and AML12 cells were used to establish *in vivo* and *in vitro* models to elucidate the effects of combined exposure to PS-MPs and Cd on the liver and their mechanisms.

**Results:**

The results showed that the combined effects of PS-MPs and Cd caused significantly more liver damage than exposure alone. As observed by transmission electron microscopy (TEM), the number of autophagosomes was significantly increased in the PS-MPs and Cd co-treated group. In addition, autophagic flux was assayed by RFP-GFP-LC3, a reporter system expressing dual fluorescent proteins, which showed an overwhelming enhancement of autophagic flux damage by co-exposure to PS-MPs and Cd compared to exposure alone. To further investigate the involvement of carnitine palmitoyltransferase1(CPT1) in liver injury induced by co-exposure to Cd and PS-MPs, we co-exposed Baicalin, an activator of CPT1, with PS-MPs and Cd, and showed that activation of CPT1 alleviated the impairment of autophagic fluxes induced by co-exposure of Cd and PS-MPs and further alleviated the changes in lipid accumulation and associated protein levels.

**Discussion:**

In conclusion, the concurrent exposure of PS-MPs and Cd resulted in the blockage of hepatic lipid accumulation and autophagic pathway and further aggravated the toxic damage to the liver. Activation of CPT1 could alleviate the PS-MPs and Cd-induced lipid accumulation and autophagy pathway blockage thus reducing liver injury.

## Graphical Abstract

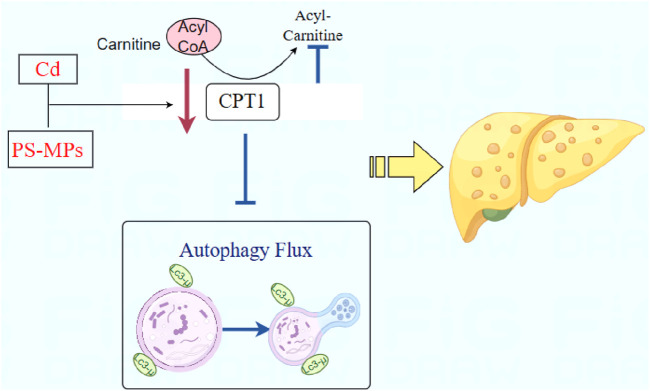

## Highlights

1. PS-MPs accumulate in mouse liver and promote Cd accumulation.
2. Co-exposure to PS-MPs and Cd exacerbate liver injury caused by exposure alone.
3. Co-exposure to PS-MPs and Cd exacerbates lipid accumulation and blockage of autophagic flux.
4. Activation of CPT1 attenuates the blockage of autophagic flux induced by co-exposure of PS-MPs and Cd, thereby reducing lipid accumulation.

## 1 Introduction

Cadmium (Cd) is a heavy metal that is ubiquitous in the environment and originates mainly from human agricultural production and industrial activities (Genchi et al., 2020). Cd is produced about 13,000 tonnes per year worldwide and is the most toxic pollutant in industrialized countries (Mężyńska et al., 2018). Currently, Cd pollution has become an unavoidable problem in the ecosystem (Zhu et al., 2018). The United Nations included Cd and its compounds in the list of carcinogens in 2017. Several studies have shown an association between Cd exposure and obesity, diabetes, and metabolic syndrome, as well as an increased risk of atherosclerosis and cardiovascular disease (Fagerberg and Barregard, 2021; Tinkov et al., 2017). In the body, the liver is the main site of Cd accumulation (Mężyńska and Brzóska, 2019). Studies have shown that Cd exposure is positively associated with the incidence of many liver diseases, including necrotizing inflammation, hyperglycemia, non-alcoholic fatty liver disease (NAFLD), and non-alcoholic steatohepatitis (NASH) (Rosales-Cruz et al., 2018). Mitochondrial β-oxidation of fatty acids is a vital pathway for fatty acid catabolism, which plays an indispensable role in regulating energy homeostasis across the entire body. CPT1 is the essential enzyme in carnitine-dependent transport across the inner mitochondrial membrane. The reduction in its quantity leads to a lower rate of beta-oxidation of fatty acid (Schlaepfer and Joshi, 2020).

Microplastics (MPs) are plastic fragments or particles less than or equal to 5 mm in diameter found in the environment. Polystyrene is a highly productive plastic and is among the most commonly occurring microplastics (MPs) in the environment (Vaid et al., 2021). It can be transported to more places by wind, snow, atmosphere, and various water currents (rivers, lakes, and oceans) (Xiang et al., 2022; Chen et al., 2020; Evangeliou et al., 2020; Luo et al., 2019a; Abbasi et al., 2019). After entering the organism, most of the MPs are excreted in the feces, especially large particles with a diameter greater than 150 μm, and smaller particles accumulate in certain organs of the body, such as the brain, heart, liver, kidneys, and epididymis (Liang et al., 2021). Current studies have shown that MPs ranging from 25 nm to 90 μm can accumulate in the liver of marine fish or mammals (rats and mice) (Babaei et al., 2022; Ding et al., 2020; Pitt et al., 2018) and are negatively correlated with MPs size (Abarghouei et al., 2021). Studies have shown that exposure of zebrafish to PS-MPs for 3 weeks resulted in hepatocellular necrosis, inflammatory infiltration, and lipid accumulation (Lu et al., 2016). Vacuolar degeneration and inflammatory infiltration were observed in mouse liver after 30 days of exposure to PS-MPs (Luo et al., 2019b). Currently, the main body of research on liver damage caused by MPs exposure is mostly marine organisms, and more experiments are needed to provide theoretical support for mammalian studies (Yin et al., 2022).

Both MPs and Cd are prevalent pollutants in the environment, and human activities have caused large amounts of Cd and MPs to enter water streams and soils, be ingested by aquatic organisms and plants, and accumulate through the food chain (Allen et al., 2021; Weber et al., 2022; Shi et al., 2016). MPs can serve as effective carriers of other pollutants in the surrounding environment, adsorbing organic pesticides, toxic heavy metals, and atmospheric pollutants (Martinho et al., 2022). Relevant researchers have detected the presence of different concentrations of heavy metals in MPs in the environment (Holmes et al., 2012). It has also been reported in the literature that adsorbed heavy metals were found in plastics in flying birds (Massos and Turner, 2017). An acute toxicity study of PS-MPs with Cd on grass carp showed that the presence of PS-MPs increased the concentration of Cd in grass carp and accelerated their death (Chen et al., 2022). A chronic toxicity experiment in zebrafish exposed to PS-MPs and Cd was consistent with its results, showing that the presence of MPs did enhance the toxicity of Cd (Lu et al., 2018a). While the effects of co-exposure of Cd and PS-MPs on the mouse liver and its mechanism are still unclear. Therefore, in this study, we used C57BL/6 mouse and AML12 cells, treated mice and cells with PS-MPs and Cd individually or in combination, explored whether PS-MPs could exacerbate Cd-induced liver damage. This study will reveal the toxicity mechanism of Cd and PS-MPs on the liver and provide a theoretical basis for the targeted prevention and treatment of Cd and PS-MPs-induced liver injury.

## 2 Materials and methods

### 2.1 Chemicals and antibodies

StubRFP-sensGFP-LC3 Lentivirus was purchased from Genechem (Shanghai, China). Cadmium chloride (CdCl_2_) was purchased from Sigma-Aldrich (St. Louis, MO, United States). Polystyrene microspheres (PS-MPs) (5 μm) (2.5%w/v, 10 mL) was purchased from BaseLine ChromTech Research Centre (Tianjin, China). Baicalin was purchased from MedChemExpress (Shanghai China). Hematoxylin and Eosin Staining Kit, Oil Red O Staining Kit, and Hoechst 33342 were purchased from Beyotime Biotechnology (Shanghai, China). BODIPY™ 493/503 was purchased from ThermoFisher Scientific (Waltham, MA, United States). ACC, CPT1, Atg7, β-actin, Beclin1, P62, and LC3 monoclonal antibody were purchased from Cell Signaling Technology (Danvers, MA, United States). Apob and Horseradish peroxidase (HRP)-conjugated goat anti-rabbit immunoglobulin G were purchased from Santa Cruz Biotechnology (CA, United States). Triglyceride (TG) assay kit, Total cholesterol (T-CHO) assay kit, and High-density lipoprotein cholesterol (HDL-C) assay kit were purchased from Nanjing Jiancheng Bioengineering Institute (Nanjing, China).

### 2.2 Experimental design

In accordance with the Yangzhou University Animal Use Protocol [IDS: SYXK (Su) 2017-0044]. This study followed previously established methods (Liang et al., 2021; Lu et al., 2016; Zhang et al., 2018), using PS-MPs obtained from BaseLine ChromTech Research Centre (Tianjin, China). Water is the main source of PS-MPs and Cd pollution, so this study is based on the concentration of Cd and PS-MPs pollution in the environment and combined with experimental requirements and previous studies. The concentrations of Cd (50 mg/L) and PS-MPs (10 mg/L) were selected (Ma et al., 2021; Prokić et al., 2021; Sun et al., 2021; Yin et al., 2023). C57BL/6J mice (6 weeks old, SPF grade) obtained from Spelford Biotechnology (Beijing, China) underwent a 1-week acclimation period. The 32 mice were randomly divided into four groups: 1) The control group [receiving double distilled water (DDW)]; 2) the PS-MPs group (administered with DDW containing 10 mg/L PS-MPs with a particle size of 5 μm); 3) the Cd group (administered with 50 mg/L Cd), and 4) the PS-MPs and Cd co-treatment group (Cd + PS-MPs, administered with purified water containing 50 mg/L Cd and 10 mg/L PS-MPs with a particle size of 5 μm. Mice were given free access to water for 3 months, during which time food intake was uncontrolled. At sacrifice by cervical dislocation, body weight was measured before the liver was removed for fixation in 10% neutral formalin or 2.5% glutaraldehyde. The relevant experiments in this study refer to previously published content (Sun et al., 2023a; Sun et al., 2023b; Wang et al., 2023) and manufacturer’s instructions.

### 2.3 Observation of the morphology of PS-MPs by scanning electron microscopy

Transfer 1 mL of PS-MPs solution to a 1.5 mL EP tube and centrifuge for 20 min at 12,000*g at 4°C. To acquire PS-MPs precipitation, dispose of the supernatant. The EP tube should then be placed in an oven at 60°C to completely evaporate the water and obtain PS-MPs powder. PS-MPs will be observed via SEM.

### 2.4 Laser confocal microscopy characterizes distribution of PS-MPs

Two male C57BL/6 mice were administered fluorescent PS-MPs via gavage individually for a duration of 1 week. After the treatment period, the mice were euthanized, and the livers were extracted to prepare frozen sections. The nuclei within the samples were stained with Hoechst 33342. Observation of the slices was done using a laser confocal microscope.

The cells were then cultured under standard conditions until the cell density reached 80%. The initial culture medium was discarded and substituted with fluorescent PS-MPs medium, which was prepared with serum-free medium. The samples were then processed for 12 h. The samples were rinsed twice with PBS and fixed using 4% paraformaldehyde for 20 min at room temperature. Following the rinse with PBS, they were incubated in Hoechst 33342 under light protection for 20 min. Finally, observation of the slices was done using a laser confocal microscope.

### 2.5 Hematoxylin and eosin staining

The samples were retained in paraformaldehyde for 24 h and subsequently trimmed. The fixed tissues were embedded in wax blocks to create paraffin sections. These sections were then dehydrated in a graded manner and stained with H&E before being washed with running tap water. The observations were made using a microscope manufactured by Leica Corporation (model number: Leica 2500) located in Wetzlar, Germany.

### 2.6 Transmission electron microscope (TEM)

Fresh liver tissue from each group was sliced into segments measuring 1–2 mm. These segments were then treated with 2.5% glutaraldehyde fixative for overnight fixation at a temperature of 4°C. Following fixation, the segments were subjected to osmic acid fixation, underwent dehydration through a graded ethanol series, and were finally embedded in the Epon 812 compound. A diamond knife was employed to cut ultrathin sections, which were negatively stained using aqueous uranyl acetate and lead citrate.

### 2.7 Cell culture and morphological observations

The AML12 cell line (American Type Culture Collection) was grown in Dulbecco’s modified Eagle’s medium (DMEM) with F-12 and 1% ITS-A (Insulin-Transferrin-Selenium-Sodium Pyruvate) (Sigma-Aldrich, St. Louis, MO, United States), supplemented with 10% fetal bovine serum (FBS), 100 U/mL penicillin, and 100 mg/mL streptomycin. The cells were maintained at 37°C under a 5% CO_2_ atmosphere. Upon achieving an initial cell density of approximately 80%, they were allocated to four groups: control group, Cd (5 μM) group, PS-MPs (10 μg/mL) group, or Cd + PS-MPs group. Cells underwent 12 h of treatment with defined media prepared from serum-free media post-discarding the existing medium. Post-washings with PBS, images were captured via microscopy observation.

### 2.8 TG, TC and HDL-C content determination

This experiment was performed according to the reagent manufacturer’s instructions. Tissue specimens were weighed, and then mixed with normal saline at a ratio of 1:9. The mixture was homogenised and centrifuged at 2500*g for 10 min before the supernatant was collected for measurement. Similarly, cell specimens were suspended in normal saline, disrupted using ultrasound, centrifuged and the supernatant was then collected for assessment. The TG, TC and HDL-C levels in the supernatants were subsequently analysed using the relevant test kits.

### 2.9 Cellular index assay

Initially, 100 µL of media was used to establish a baseline in each well. Following this stage, 100 μL of cell suspension (containing an appropriate amount of cells) was added per well, vortexed, and introduced into our detection apparatus. The cells were left undisturbed for a period of 15 min before the start of Cellular index (CI) monitoring, which occurred automatically every 15 min. Once the cell density exceeded 80%, subsequent treatments were initiated while maintaining CI monitoring and normalization.

### 2.10 Oil red O staining

This experiment was performed according to the reagent manufacturer’s instructions. Cells were seeded into 6-well plates and treated when cell density reached around 80%. The initial medium was removed, and the necessary concentration of culture medium pre-prepared with serum-free medium was added for 12 h. After removing the medium, PBS was used to rinse the wells once, and 1 mL of staining wash solution was added to each one and left for 20S. The wash solution was removed, and each well was treated with 1 mL of modified oil red O staining solution for 30 min. After completing the staining process, the wash solution was added and left for 30S followed by its disposal. Subsequently, the wash solution was added again and left for 20S. The cells were then washed using PBS for a duration of 20S. Next, PBS was added to each well, ensuring even coverage of the cells. Finally, the samples were placed under a microscope for observation and photography.

### 2.11 BODIPY staining

This experiment was performed according to the reagent manufacturer’s instructions. The samples were prepared in 24-well plates and maintained under standard conditions. Once the cellular density reached 80%, treatments were initiated. The initial media was removed and replaced with pre-prepared culture medium devoid of serum, and left for 12 h. The cells were washed twice with PBS, fixed with 4% paraformaldehyde for 20 min. Next, each well was washed with PBS once before introducing BODIPY 493/503 dye dilution. The dye was added at a volume of 400 μL per well, followed by incubation at 37°C, shielded from light, for 15 min. Subsequently, a PBS wash was done followed by a BODIPY stain rinse, both at room temperature. Another PBS wash was performed immediately before proceeding with Hoechst 33342 incubation for 20 min, also at room temperature. After another wash in PBS, the sections, which had been stained, were sealed for analysis using confocal microscopy.

### 2.12 Western blotting analysis

Tissue suspensions were homogenised and transferred to a 1.5 mL centrifuge tube. Subsequently, the tube was subjected to centrifugation at 1000*g for 10 min resulting in the initial supernatant. This supernatant was then further centrifuged at 12,000*g for 15 min, yielding the final supernatant. Concerning cell samples, cells were harvested from dishes and were lysed with RIPA buffer for 30 min at 4°C. Further, ultrasonification was performed, and the sample was subjected to centrifugation at 12,000*g for 15 min. Protein concentrations were determined through Bicinchoninic acid assays (YeasenBio Technology, Shanghai, China). Each sample was supplemented with a ×5 loading buffer in a 1:4 ratio and subsequently underwent high temperature denaturation for 10 min. Subsequently, the proteins were electrophoresed on sodium dodecyl sulfate-polyacrylamide gels and transferred onto polyvinylidene difluoride membranes (Millipore, MA, United States). Following blocking and washing with TBST, the membranes were exposed to primary and secondary antibodies. Band density quantification was conducted with the aid of Image Lab software (Bio-Rad, CA, United States).

### 2.13 Statistical analysis

The data from at least three independent experiments were analyzed statistically and expressed as the mean ± standard deviation (SD). GraphPad Prism 6 software (GraphPad Software Inc., La Jolla, CA, United States) was used to analyze the data using one-way analysis of variance [(ANOVA) (Scheffe’s SF test)]. *P* values less than 0.05 indicated a significant difference.

## 3 Results

### 3.1 PS-MPs accumulate in mouse liver and promote Cd accumulation

**FIGURE 1:**
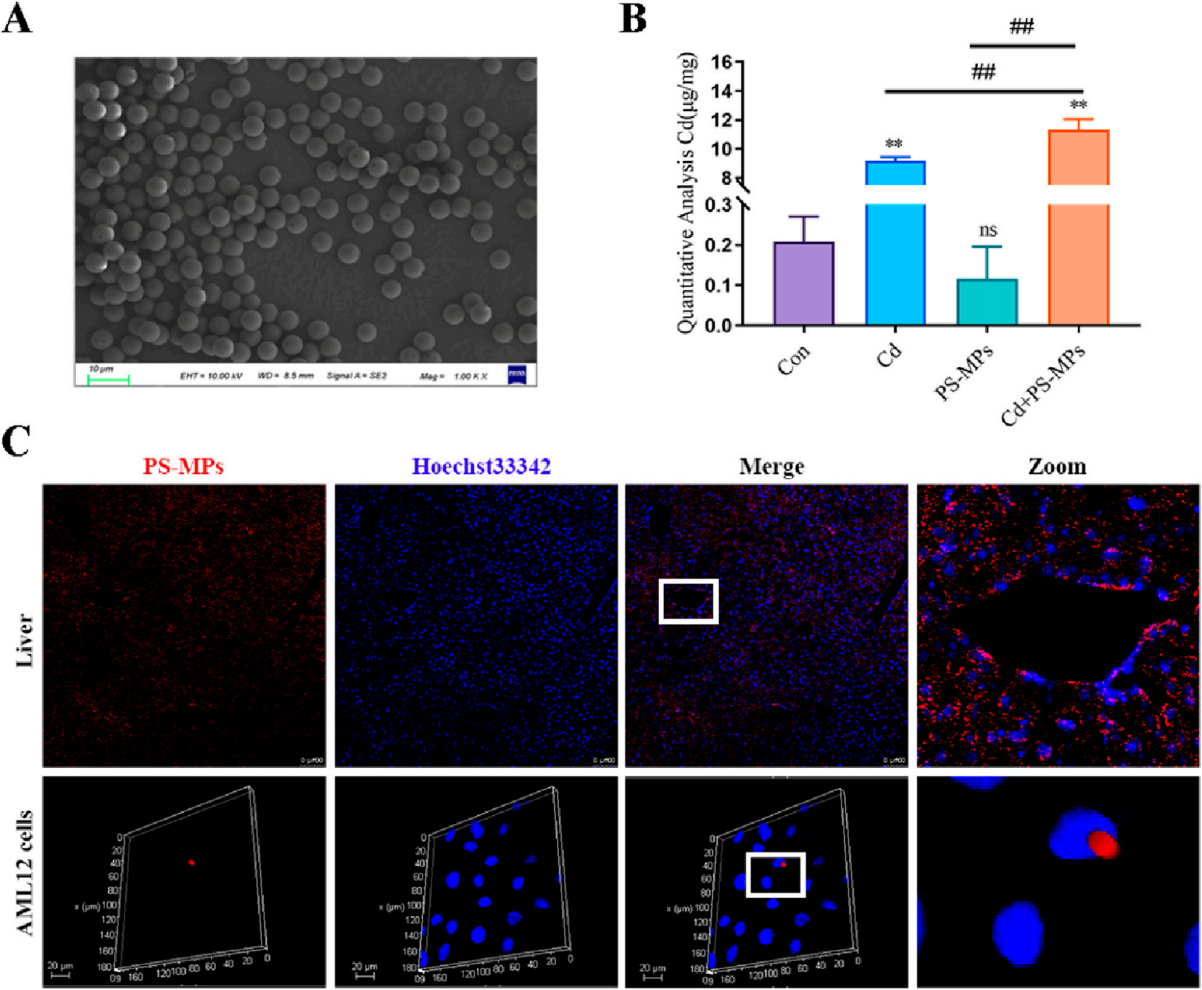
PS-MPs accumulate in mouse liver and promote Cd accumulation. Morphology of PS-MPs **(A)**. The concentration of Cd in the liver **(B)**. Levels of PS-MPs in the li ver and AML12 **(C)**. Scale bar = 100 μm, Scale bar = 20 μm. Results are shown as the mean ± SD (n = 3). Compared with the control group, *P < 0.05, **P < 0.01. Compared with the Cd + PS-MPs group, ^#^ *P* < 0.05, ^##^ *P* < 0.01.

Firstly, we observed the morphology of the PS-MPs using SEM, and the results, as shown in Figure 1A, showed that the PS-MPs were of uniform particle size, with smooth surfaces and regular spherical shapes. In order to further investigate the effect of co-exposure of PS-MPs and Cd on the Cd accumulation profile, Cd levels in the liver were examined in this study, and the results are shown in Figure 1B. Compared to the control group, the Cd content within the Cd group was significantly increased, and when PS-MPs and Cd were co-exposed, it further increased the accumulation of Cd. Subsequently, we also further examined the accumulation of PS-MPs in the liver, and the results, as shown in Figure 1C, showed that the red fluorescence carried by PS-MPs was observed in both the liver and AML12.

### 3.2 PS-MPs and Cd co-exposure exacerbate liver injury induced by exposure alone

**FIGURE 2:**
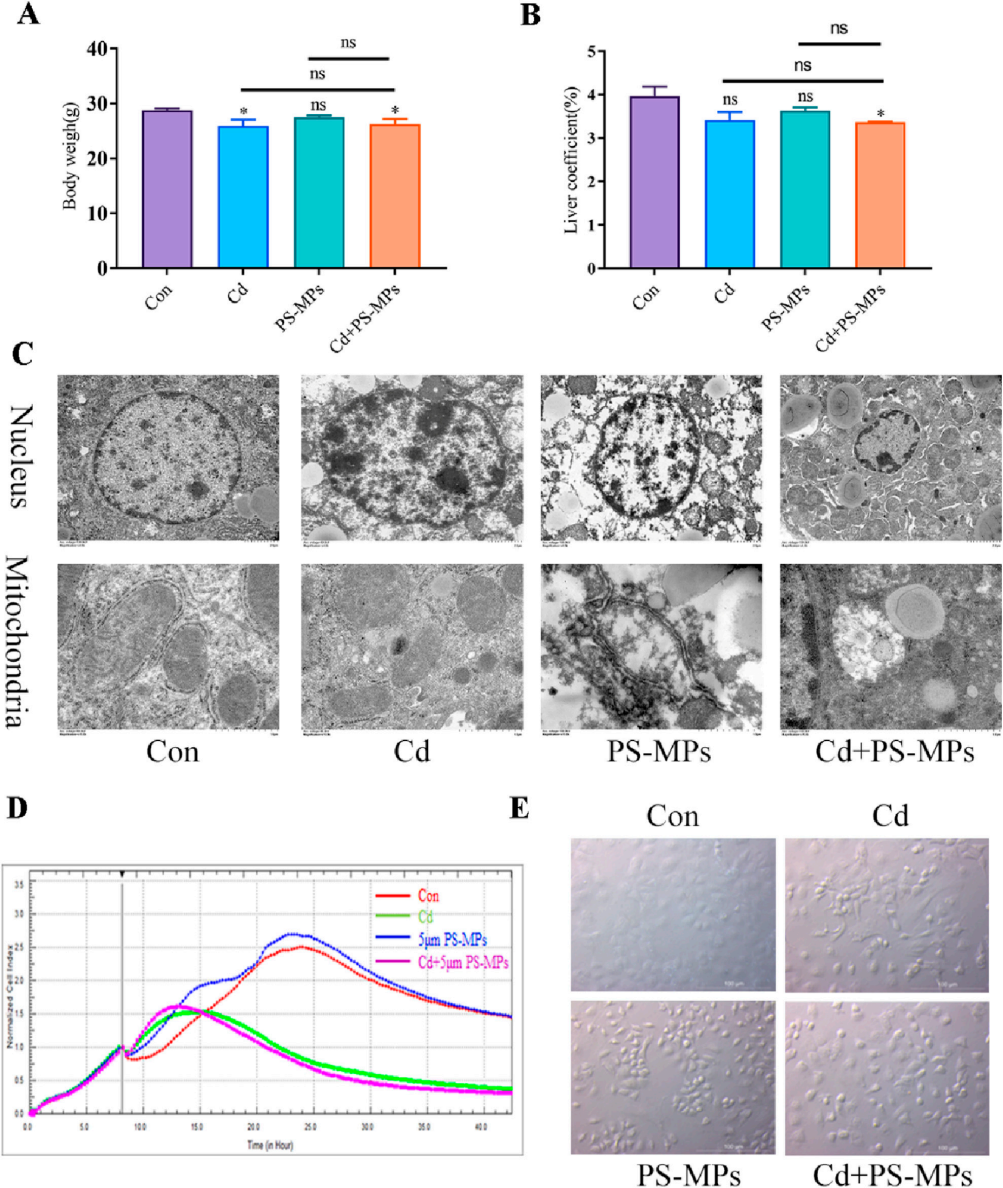
PS-MPs and Cd co-exposure exacerbates liver injury induced by exposure alone. Body weights **(A)** and the liver coefficient **(B)** in mice. Transmission Electron Microscope (TEM) observed the ultrastructure of nucleus and mitochondria **(C)**. Scale bar = 2 μm, Scale bar = 0.5 μm. AML12 cells were observed after exposure of Cd and PS-MPs for 12 h. Cells index was detected by RTCA **(D)**. Brightfield observation of cell morphology **(E)**. Scale bar = 100 μm. Results are shown as the mean ± SD (n = 3). Compared with the control group, *P < 0.05, **P < 0.01. Compared with the Cd + PS-MPs group, ^#^ *P* < 0.05, ^##^ *P* < 0.01.

This study began by measuring changes in body weight and liver coefficient. The results revealed that the Cd and Cd + PS-MPs groups experienced a decrease in body weight compared to the control group. However, there were no changes observed between the Cd + PS-MPs group and the Cd group. Exposure to Cd or PS-MPs did not elicit a significant impact on the liver coefficient; however, their combined exposure resulted in a reduction (Figures 2A, B). Subsequently, TEM was implemented to examine the ultrastructure of hepatocytes. The control group displayed distinct nuclei with regularly distributed chromatin. Conversely, the treated group experienced nuclei morphology variation, intensified nuclear chromatin staining, constricted nuclear membrane surrounding the nucleus, and an amplified nuclear pore gap. The Cd + PS-MPs group exhibited more modifications in nuclear morphology, greater nuclear crumpling, and increased intercellular gaps in comparison to the Cd group. On the other hand, the control group showed normal mitochondria structure with well-defined internal and external membranes along with a uniform distribution of cristae. The mitochondria observed in the Cd group appeared to be swollen, deformed, and had a blurred bilayer structure. Furthermore, the mitochondrial cristae in the Cd + PS-MPs group exhibited fractures, dissolution, and vacuolization to a greater extent than those in the Cd group (Figure 2C). We established an *in vitro* model using AML12 cells. The cells were treated with Cd and PS-MPs either independently or in combination. We utilized RTCA technology to obtain real-time measurements of the Cellular index (CI). The findings, as presented in Figure 2D demonstrate that the CI was achieved by the Cd group and Cd + PS-MPs group after 12 h of exposure, followed by a subsequent decline. On the contrary, neither the control group nor the PS-MPs group displayed a decrease in CI until approximately 24 h of exposure. The cells were observed through bright field conditions. The results are presented in Figure 2E, in comparison to the control group, the treated group exhibited indications of wrinkling, wider gaps, and clear alterations in their morphology. Moreover, the cell density was notably reduced in both the Cd group and the PS-MPs group. Overall, co-exposure to PS-MPs and Cd exacerbates liver injury by disrupting hepatocyte morphology and function.

### 3.3 Co-exposure of Cd and PS-MPs disrupts lipid metabolism and thus exacerbates lipid accumulation

**FIGURE 3:**
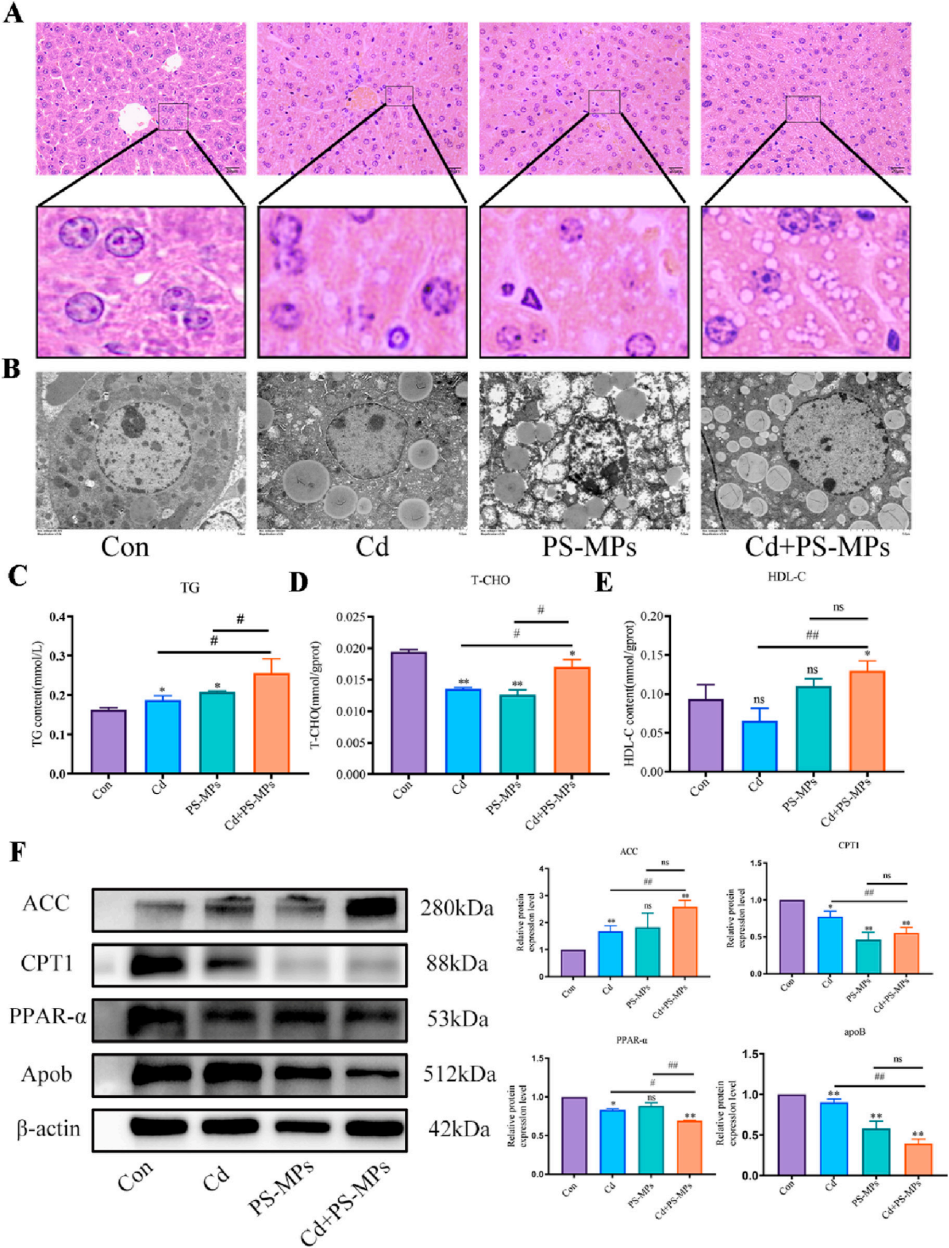
Co-exposure to Cd and PS-MPs exacerbates lipid accumulation in mice. Liver histopathology observed using HE staining **(A)**. Scale bar = 20 μm. TEM observed the ultrastructure of lipid droplets **(B)**. Scale bar = 5 μm. TG **(C)**, T-CHO **(D)**, and HDL-C **(E)** levels in the mice were measured. Levels of ACC, CPT1, PPAR-α, and Apob were determined using Western blotting **(F)**. Results are shown as the mean ± SD (n = 3). Compared with the control group, *P < 0.05, **P < 0.01. Compared with the Cd + PS-MPs group, ^#^ *P* < 0.05, ^##^ *P* < 0.01.

**FIGURE 4:**
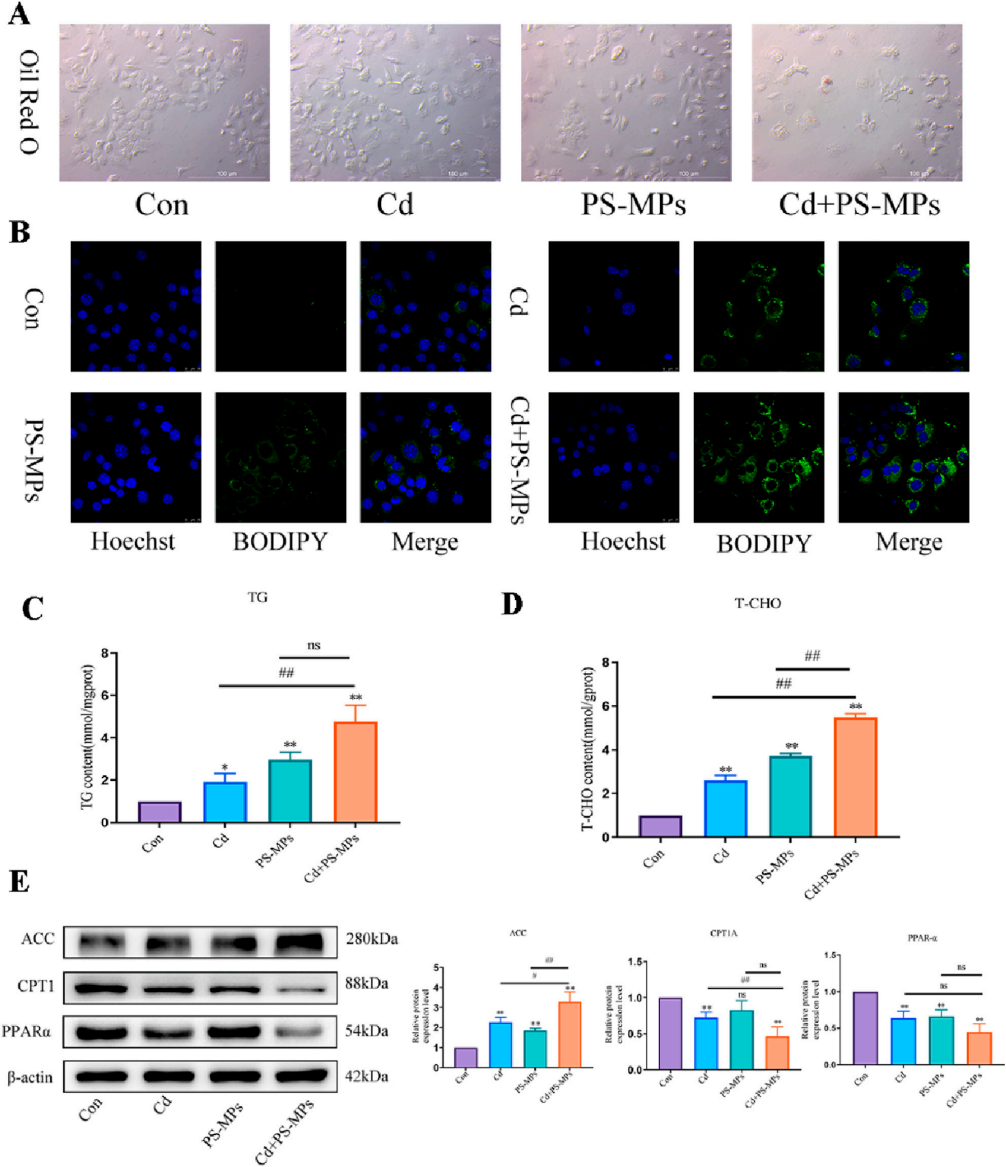
Co-exposure to Cd and PS-MPs exacerbates lipid accumulation in AML12. Oil red O staining **(A)** to and BODIPY staining **(B)** observe the content of neutral fat. Scale bar = 100 μm. Scale bar = 25 μm. TG **(C)** and T-CHO **(D)** levels in the AML12 were measured. Levels of ACC, PPAR-α, and CPT1 were determined using Western blotting **(E)**. Results are shown as the mean ± SD (n = 3). Compared with the control group, *P < 0.05, **P < 0.01. Compared with the Cd + PS-MPs group, ^#^ *P* < 0.05, ^##^ *P* < 0.01.

Figure 3A depicts the HE staining results. White, rounded lipid droplets were observed in both the Cd group and the PS-MPs group, either alone or in the combined exposure group. These findings are consistent with observations obtained via TEM (Figure 3B). The kits detected T-CHO, TG, and HDL-C content in the liver tissue of mice. Figures 3C–E presents the results. Compared to the control group, T-CHO content significantly decreased in each treatment group. TG content significantly increased in each treatment group. HDL-C notably increased in the Cd + PS-MPs group and showed a tendency to rise in other groups but with no statistical variance. The lipid metabolism-related proteins were identified by WB. The ACC exhibited a significant increase in the Cd group relative to the control group. Furthermore, in comparison with the Cd group, the Cd + PS-MPs group displayed a significant elevation in ACC levels. The levels of PPAR-α, CPT1, and Apob exhibited a declining trend among all treatment groups as compared to the control group. Moreover, the expression of PPAR-α, CPT1, and Apob was reduced in the Cd + PS-MPs group in contrast to the Cd group (Figure 3F). The experiment also revealed oil red O staining in AML12 cells, confirming lipid droplet accumulation, as illustrated in Figure 4A. A few orange lipid droplets were visible in the control group, while the Cd or PS-MPs groups had increased amounts of droplets compared to the control group. Additionally, the Cd + PS-MPs group had more lipid droplets. Lipid droplets were detected through fluorescent staining in AML12 cells, as illustrated in Figure 4B. Comparison to the control group, the Cd or PS-MPs group exhibited an increased number of green aggregates along with enhanced fluorescence intensity. The Cd + PS-MPs group demonstrated a further increase in the number of green aggregates and their fluorescence intensity in comparison to the Cd or PS-MPs groups. The kits detected T-CHO and TG content in the AML12 cells. Figures 4C, D displays the results. Compared to the control group, all treatment groups had significantly higher levels of T-CHO and TG. The Cd + PS-MPs group demonstrated a significant increase in T-CHO and TG content compared to the Cd group. Changes in the expression of ACC, CPT1, and PPAR-α showed consistency with the *in vivo* experiments (Figure 4E). The co-exposure of Cd and PS-MPs has been identified as a key factor in the promotion of lipid accumulation, with the inhibition of CPT1 expression emerging as a key mechanism through which this occurs.

### 3.4 Combined exposure to Cd and PS-MPs exacerbates exposure alone-induced blockage of autophagic flux

**FIGURE 5:**
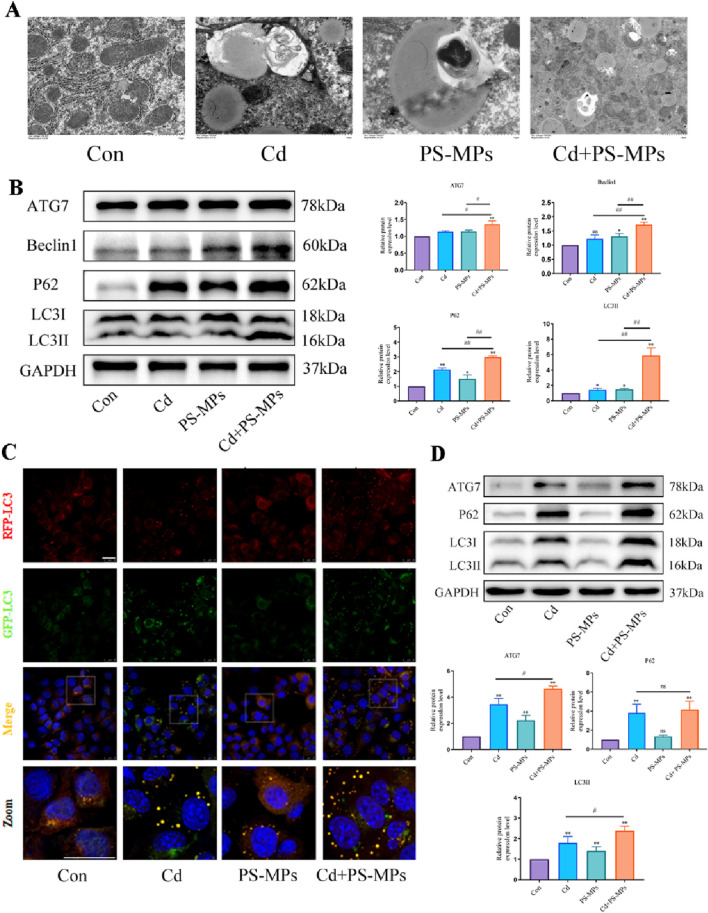
Co-exposure to Cd and PS-MPs exacerbates exposure alone-induced blockage of autophagic flux. TEM observation of hepatocyte autophagosomes **(A)**. Scale bar = 2 μm. Levels of ATG7, Beclin1, P62, and LC3 were determined using Western blotting **(B)**. AML12 cells were observed after exposure of Cd and PS-MPs for 12 h. Representative images of RFP-GFP-LC3 puncta **(C)**. Scale bar = 25 μm. Levels of ATG7, P62, and LC3 were determined using Western blotting **(D)**. Results are shown as the mean ± SD (n = 3). Compared with the control group, *P < 0.05, **P < 0.01. Compared with the Cd + PS-MPs group, ^#^ *P* < 0.05, ^##^ *P* < 0.01.

TEM examination revealed the presence of autophagosomes in mouse liver as illustrated in Figure 5A. In comparison to the control group, autophagosomes featuring a double-membrane structure coalesced with lipid droplets were noted in the treatment group. There were higher incidences of autophagosomes in the Cd + PS-MPs group than in those treated with Cd or PS-MPs. In comparison to the control group, there was a tendency for an increment in the expression of P62, LC3, ATG7, and Beclin1 in all treatment groups. When compared to the Cd group, the expression of P62, LC3, ATG7, and Beclin1 increased significantly in the Cd + PS-MPs group (Figure 5B). Autophagic flux detection using RFP-GFP-LC3 in AML12. The results indicate an increase in yellow fluorescent aggregates in the Cd group along with their brightness, while denser yellow aggregates emerged in the PS-MPs group compared to the control group. More and brighter yellow fluorescent aggregates were observed in the Cd + PS-MPs group in comparison to the Cd group (Figure 5C). The results are showcased in Figure 5D. The expression of P62, LC3, and ATG7 was extremely increased in all treatment groups when compared to the control group. Compared to the Cd group, the expression of LC3 and ATG7 was higher in the Cd + PS-MPs group. Additionally, the expression of P62 showed a tendency to increase, although this was not statistically significant.

### 3.5 Activation of CPT1 alleviates PS-MPs and Cd co-exposure-induced blockage of autophagic flux

**FIGURE 6:**
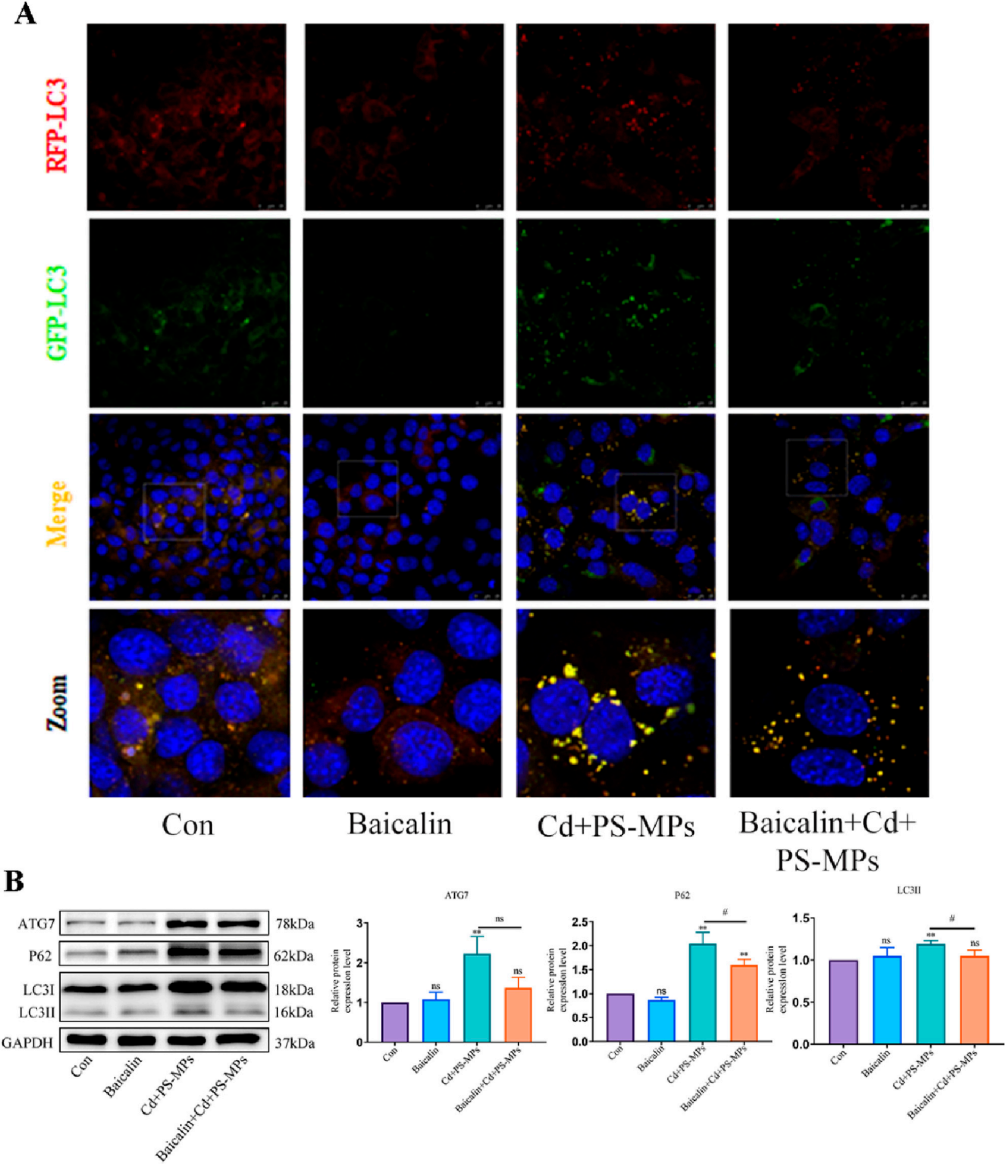
Activation of CPT1 alleviates PS-MPs and Cd co-exposure- induced blockage of autophagic flux. Representative images of RFP-GFP-LC3 puncta **(A)**. Scale bar = 25 μm. Levels of ATG7, P62, and LC3 were determined using Western blotting **(B)**. Results are shown as the mean ± SD (n = 3). Compared with the control group, *P < 0.05, **P < 0.01. Compared with the Cd + PS-MPs group, ^#^ *P* < 0.05, ^##^ *P* < 0.01.

The results are presented in Figure 6A. It was observed that the control and Baicalin groups had minimal yellow aggregates, whereas the yellow aggregates in the Cd + PS-MPs group increased in number and brightness. The yellow aggregates in the Baicalin + Cd + PS-MPs group were significantly reduced. In comparison to the control group, the expression of ATG7, P62, and LC3 was higher in the Cd + PS-MPs group. Conversely, in the Baicalin + Cd + PS-MPs group, the expression of ATG7, P62, and LC3 was significantly lower in comparison with the Cd + PS-MPs group (Figure 6B).

### 3.6 Relief of autophagic flux blockade reduces liver lipid accumulation

**FIGURE 7:**
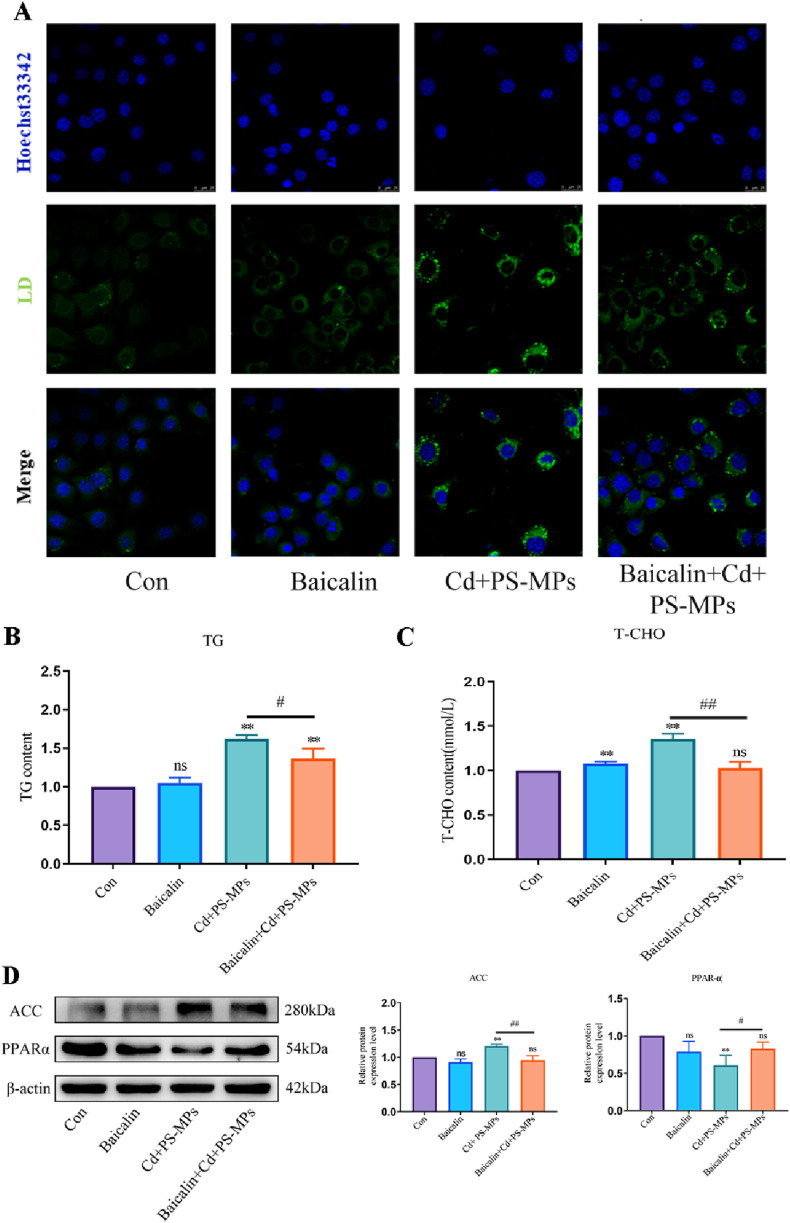
Activation of CPT1 alleviates PS-MPs and Cd co-exposure-induced lipid accumulation. BODIPY staining observe the content of neutral fat **(A)**. Scale bar = 25 μm. TG **(B)** and T-CHO **(C)** levels in the AML12 were measured. Levels of ACC and PPAR-α were determined using Western blotting **(D)**. Results are shown as the mean ± SD (n = 3). Compared with the control group, *P < 0.05, **P < 0.01. Compared with the Cd + PS-MPs group, ^#^ *P* < 0.05, ^##^ *P* < 0.01.

In the Baicalin + Cd + PS-MPs groups, 100 μmol/L baicalin was added beforehand followed by exposure to Cd and PS-MPs for 12 h after 1 h. The outcomes were exhibited in Figure 7A. The results indicate that the number of lipid droplets was minimal in both the control and Baicalin groups, in comparison to a significant increase in lipid droplets observed in the Cd + PS-MPs group. The Baicalin + Cd + PS-MPs group exhibited a considerable decrease in lipid droplet content relative to the Cd + PS-MPs group. The content of T-CHO and TG was identified, with the outcomes presented in Figures 7B, C. The levels of T-CHO and TG were increased in the Cd + PS-MPs group compared to the control group. Conversely, the Baicalin + Cd + PS-MPs group displayed a significant decrease in T-CHO and TG levels relative to the Cd + PS-MPs group. When compared to the control group, the Cd + PS-MPs group displayed a notable rise in ACC and a decrease in PPAR-α expression. Additionally, when compared to the Cd + PS-MPs group, the Baicalin + Cd + PS-MPs group exhibited an increase in PPAR-α expression and a decrease in ACC expression (Figure 7D). Overall, restoration of autophagic flux alleviated lipid accumulation induced by co-exposure to PS-MPs and Cd.

## 4 Discussion

Cd pollution is a significant public health concern on a global scale (Wang et al., 2015). Cd can be found in a range of sources, such as water, soil, food, and tobacco products, and can enter the body via multiple pathways. The increasing worldwide production of MPs has generated significant apprehension over their potential contamination, and their impact on animal health has drawn attention across the world (Chen et al., 2021; Horton et al., 2017). MPs are ubiquitous in the air, soil, water, and many food and household items. They penetrate the organism through the digestive, respiratory, and dermal routes, causing harm (Yin et al., 2022). PS-MPs is a highly prolific plastic and one of the prevailing MPs types observed in the environment (Lu et al., 2016). The liver is a crucial detoxifying organ in mammals and is sensitive to several toxic agents (Mężyńska et al., 2018; Saïdi et al., 2013). Studies have demonstrated that PS-MPs and Cd frequently target the liver (Mężyńska and Brzóska, 2019; Goodman et al., 2022). The current study demonstrated that PS-MPs intensified the accumulation of Cd in the liver of mice, aligning with the findings of Lu et al. (2018a)’s zebrafish tissue experiment on the impact of PS-MPs on Cd accumulation. Fluorescent PS-MPs were orally administered to mice, and subsequent liver sections were examined. Results revealed that PS-MPs accumulated in the liver with a more uniform distribution. Likewise, exposure of AML12 cells to PS-MPs for 12 h displayed the presence of PS-MPs in the cells, mainly concentrated around the nucleus. These findings demonstrate the accumulation of PS-MPs in the liver cells. The findings indicate that PS-MPs and Cd are capable of entering hepatocytes, providing a basis for further investigation into the toxicity mechanism of PS-MPs and Cd. The study also analyzed the influence of Cd and PS-MPs on mouse weight and liver index revealing significant reductions post Cd exposure, following Brzoska’s report (Brzóska et al., 2003). The body weight of mice was significantly reduced following exposure to PS-MPs, which is in line with Lu et al. (2018b) and Jin et al. (2021)’s findings. Although exposure to PS-MPs alone had no statistically significant effect on the liver coefficient of mice, it is noteworthy that the Cd + PS-MPs group had the lowest liver coefficient. This suggests that the joint action of Cd and PS-MPs on the liver resulted in a greater toxicity of Cd, while simultaneously worsening the liver damage through the intensification of the PS-MPs. This led to necrosis and deformation of certain liver regions, ultimately culminating in a reduction of the liver coefficient. The HE staining observations of pathological sections of the liver and TEM observations of the ultrastructure of liver cells confirmed the damage inflicted by exposure to Cd and PS-MPs. The bodily harm includes liver cord ailments, nucleus crumpling, mitochondrial cristae rupture, and vacuolisation.

The liver regulates lipid homeostasis by absorbing fatty acids, undergoing *de novo* lipogenesis, producing triglycerides, and dividing fatty acids (Jones, 2016). The influence of Cd exposure on liver lipid metabolism remains insufficiently researched. In their examination of Cd-exposed zebrafish, Pan et al. (2018) noted that Cd-induced oxidative stress and mitochondrial malfunction resulted in liver lipid accumulation. Wu et al. (2017) found that Cd concentrations at 200 μg/L and 500 μg/L can impact the lipid metabolism of toad liver. Furthermore, Cd can also disrupt the liver’s lipid metabolic homeostasis in animal models involving mice and rats, leading to lipid accumulation in the liver (Zhang et al., 2018; Go et al., 2015). Research into the effects of PS-MPs exposure on liver lipid metabolism is currently limited. Lu et al. (2016) observed the accumulation of PS-MPs in the livers of zebrafish after 7 days of exposure, leading to disruption of liver lipid and energy metabolism. Similar findings were made by Lu et al. (2018b) and Deng et al. (2017). Cheng and colleagues researched the harmful effects of PS-MPs on the human liver with the aid of LOs, which are liver-like organs produced by stem cells, as a substitute model. Their conclusions revealed that PS-MPs impeded the expression of genes and protein synthesis of PPARα, while increasing lipid accumulation in LOs (Cheng et al., 2022). In this experimental study, we found that Cd and PS-MPs exposure significantly increased the content of TG in liver tissues, and PS-MPs exacerbated the Cd-induced TG elevation. The above results suggest that Cd and PS-MPs exposure caused abnormal lipoprotein metabolism, which led to lipid accumulation in the liver, and PS-MPs exacerbated the lipotoxicity of Cd on the liver to a certain extent. The results of HE staining and TEM observation of the ultrastructure also proved that. ACC and FAS are key proteins for lipid synthesis. β-oxidation is the main pathway for fatty acid consumption by liver, and the main process was regulated by PPARα regulation, and CPT1 assists fatty acyl coenzyme A to enter the mitochondria and is a key enzyme in β-oxidation (Alves-Bezerra and Cohen, 2017). The results of the present study showed that exposure to Cd and PS-MPs elevated the expression of ACC and decreased the expression of PPARα, CPT1, and Apob, and the presence of PS-MPs exacerbated the Cd-induced abnormalities of lipid metabolism, which was in accordance with the previous results. Elevated expression of ACC further inhibited the expression of CPT1 and suppressed the rate of β-oxidation, which resulted in the ability of lipid degradation within the liver to be Reduced. Oil red O staining, lipid droplet fluorescence staining, T-CHO, TG and lipid metabolism-related proteins were performed on AML12 cells, and the results were consistent with vivo studies.

Autophagy is a cellular regulatory mechanism, present naturally, which preserves the stability of the intracellular environment by breaking down toxic components and damaged organelles (Niture et al., 2021). Several experimental studies on the Cd-induced autophagy in liver have been performed, demonstrating the enhancement of autophagy levels and hindrance of autophagic flux in liver (Duan et al., 2023) (Sun et al., 2022). In a survey conducted on freshwater fish, it was discovered that PS-MPs elicit autophagy and apoptosis via disruption of cellular signaling. Zhong et al. (2022) revealed that the co-exposure to arsenic and nanoplastics induces excessive autophagy by modifying the levels of PI3K, mTOR, Beclin1, ATG5, LC3, and P62 expression. In this study, TEM analysis revealed the presence of autophagic vesicles in the liver tissues of groups exposed to Cd and PS-MPs. These vesicles were found near or involved in the degradation of lipid droplets, consistent with previous findings by Singh et al. (2009) regarding the involvement of autophagy pathways in lipid droplet metabolism. There is a significant rise in the expression of ATG7, P62, Beclin1, and LC3 in the groups exposed to Cd and PS-MPs, as suggested by the findings. Later, the effects of Cd and PS-MPs on autophagic flux were detected. Yellow fluorescent aggregates were observed in Cd and PS-MPs groups. The yellow aggregates of the Cd + PS-MPs group were brighter, indicating a more severe blockage of autophagic flux. In summary, the exposure to cadmium and PS-microplastics resulted in the blockage of autophagic flux in mouse liver and AML12 cells. Furthermore, PS-MPs exacerbated the Cd-induced blockage of autophagic flux.

The main pathway for fat depletion is through fatty acid β-oxidation in mitochondria, which is divided into three main stages: activation of fatty acids, transfer of lipoyl coenzyme A and β-oxidation of lipoyl coenzyme A (Houten et al., 2016). The long-chain lipoyl coenzyme A produced by esterification in the cytosol in the first stage has to enter the mitochondrial matrix for oxidation, at which point CPT1 is required to mediate it (Neess et al., 2015). CPT1 is therefore the key rate-limiting enzyme for fatty acid β-oxidation (Bonnefont et al., 2004). It has been shown that increasing CPT1 activity effectively enhances the rate of β-oxidation and improves lipid accumulation in the liver (Dai et al., 2018). Lipid metabolism also affects autophagy. Mikami et al. (2012) induced mice with a high-fat diet and observed an increase in autophagic vesicles and a decrease in autophagic flux in WAT of obese mice. Similar conclusions were reached that an abnormal increase in lipids leads to blockage of autophagic flux (Xie et al., 2020). This interrelationship can trap hepatocytes in a deleterious cycle whereby lipid accumulation affects autophagy and autophagy inhibition further affects lipid degradation, leading to lipid accumulation in the liver. Therefore, we postulated whether CPT1 could impact autophagy and ultimately reduce lipid accumulation. Baicalin was utilized to activate CPT1, while AML12 cells underwent exposure to Cd and PS-MPs for 12 h in this investigation. Findings showed that the addition of baicalin significantly reduced lipid accumulation in the group. Activation of CPT1 by baicalin resulted in a marked reduction in the expression of ACC as well as a significant increase in the expression of PPARα, indicating a potential acceleration of β-oxidation. The expression of P62 and LC3 significantly decreased. The RFP-GFP-LC3 tandem fluorescent protein assay results presented a significant reduction of yellow fluorescent spots after the addition of baicalin. This suggests that there was smooth fusion between autophagosomes and lysosomes, and fluent autophagic flux.

## 5 Conclusion

Overall, both Cd and PS-MPs enter and accumulate in mouse and AML12 cells. Exposure to Cd and PS-MPs, alone or in combination, resulted in significant decreases in body weight and liver coefficients in mice. Exposure to PS-MPs exacerbated Cd liver damage to some extent. Exposure to Cd or PS-MPs increased fatty acid production and impeded lipid metabolism, leading to atypical lipid accumulation. PS-MPs exacerbated Cd-induced lipid accumulation. In combination with Cd and PS-MPs, CPT1 is a key target for inducing lipid accumulation. Activation of CPT1 significantly increased the β-oxidation of fatty acids and alleviated the blockage of autophagic flux, thereby reducing the hepatotoxicity induced by the combined exposure to Cd and PS-MPs. This study demonstrated the mechanism of hepatotoxicity induced by Cd and PS-MPs, and provided a new theoretical basis for the prevention and treatment of liver injury and lipid accumulation.

## Data Availability

The original contributions presented in the study are included in the article/supplementary material, further inquiries can be directed to the corresponding author.
